# Missing gene identification using functional coherence scores

**DOI:** 10.1038/srep31725

**Published:** 2016-08-24

**Authors:** Meghana Chitale, Ishita K. Khan, Daisuke Kihara

**Affiliations:** 1 Department of Computer Science, Purdue University, West Lafayette, IN, 47907, USA; 2 Department of Biological Sciences, Purdue University, West Lafayette IN, 47907, USA

## Abstract

Reconstructing metabolic and signaling pathways is an effective way of interpreting a genome sequence. A challenge in a pathway reconstruction is that often genes in a pathway cannot be easily found, reflecting current imperfect information of the target organism. In this work, we developed a new method for finding missing genes, which integrates multiple features, including gene expression, phylogenetic profile, and function association scores. Particularly, for considering function association between candidate genes and neighboring proteins to the target missing gene in the network, we used Co-occurrence Association Score (CAS) and PubMed Association Score (PAS), which are designed for capturing functional coherence of proteins. We showed that adding CAS and PAS substantially improve the accuracy of identifying missing genes in the yeast enzyme-enzyme network compared to the cases when only the conventional features, gene expression, phylogenetic profile, were used. Finally, it was also demonstrated that the accuracy improves by considering indirect neighbors to the target enzyme position in the network using a proper network-topology-based weighting scheme.

Genome sequences are now routinely determined in biology labs using the recent fast sequencing technology. Interpretation of a determined genome sequence, including function annotation of individual genes in the genome, is a crucial, fundamental step for deducing biological information embedded in the sequence. Substantial efforts have been paid in recent years to develop gene function prediction methods that can provide more effective accurate function annotation^1^,^2^,^3^. As a systematic interpretation of a genome, metabolic and signaling pathways can be constructed with annotated proteins in the genome. This step, called pathway (or network) reconstruction^4^,^5^,^6^,^7^, provides a two-dimensional structure and biological context from the one-dimensional genome sequence, leading to the possibility of computational simulations of pathways^8^,^9^,^10^. However, pathway reconstructions is inherently incomplete as it reflects the current imperfect information of the organism. There are two types of missing information in pathway reconstructions: The first type is a reaction that is missing in a network, which leads to a dead-end of production or excessive consumption of metabolites^11^,^12^,^13^. The second type is a reaction that is known to exist in the target organism but the gene that encodes the enzyme that carries out the reaction is not identified yet. Such reactions are called orphan reactions and the problem to identify the corresponding enzymes is called the missing enzyme problem^14^,^15^. It is estimated that enzymes for over 20% of known reactions are still missing^16^,^17^,^18^. Orphan reactions are classified into global, where no genes for that reaction are found in any organisms, and local, where a corresponding gene is not found in an organism of interest^15^.

A main reason that local orphan reactions occur is that the corresponding gene in the organism has substantially diverged from known sequences of the enzyme in other organisms for sequence similarity-based techniques^19^,^20^,^21^ to identify it. Thus, to complement sequence-based assignments, methods have been developed that use genomic or proteomic contexts of genes for identifying missing proteins. The underlying idea behind these methods is that the missing gene in the network tend to have a similar context to neighboring enzymes in the network. Context features used include gene expression data^22^,^23^,^24^, comparative genomics features such as gene fusion, conserved gene order, and phylogenetic profiles^25^,^26^,^27^,^28^. Phylogenetic profile and conserved gene order information are combined in a method proposed by Yamanishi *et al*.^29^. Chen *et al*. used a combination of sequence similarity, common operon structure, and phylogenetic profile to identify missing genes^30^. Gene order, gene fusion events, phylogenetic profile, gene expression, and protein-protein interaction were combined as features in a machine learning framework by Church and his colleagues^31^.

At this juncture, it would be appropriate to discuss the relationship of the missing gene identification with general gene function prediction. They are similar in the sense that both of them map a protein to biological function and in techniques used for the mapping. However, the direction of the mapping is different between the two. In regular protein function prediction, input data is a representation of a target protein, typically the amino acid sequence^32^,^33^,^34^,^35^ or the tertiary structure of the protein^36^,^37^,^38^, and the output is predicted biological function of the protein. There are function prediction methods that consider multiple different features of a target protein^39^,^40^,^41^ but still the logical flow of such methods is the same. In contrast, in the missing gene identification, input is the biological context (e.g. the name of the enzyme) of the missing gene, and output is prediction of a gene in a target genome, which is predicted to be the missing gene. Thus, the logical flow of the missing gene identification is opposite from the regular protein function prediction method, because a method takes biological function as input and output a gene that fits to the function. Another difference is that the missing gene problem occurs specifically when pathways are reconstructed from genes in a genome. Thus, information of neighboring genes in the pathway is available. On the other hand, general protein function prediction usually needs to predict its function only from the information of the single protein.

In this work, we developed a new method named GO-MEP (Gene Ontology-based Missing Enzyme Predictor) for finding missing genes that integrates multiple features, including gene expression, phylogenetic profile, and function association scores developed in our group. Particularly, for considering function association between candidate genes and neighboring proteins to the target missing gene in the network, we used Co-occurrence Association Score (CAS) and PubMed Association Score (PAS), which were developed in our previous work^42^. The two scores are designed for capturing *functional coherence,* rather than simple functional similarity of proteins, and aim to identify proteins that play coherent roles in the same functional unit. CAS and PAS were computed from the frequency of GO term pairs used to annotate individual genes and those which occur in the same PubMed abstracts, respectively. Compared to existing GO term similarity scores developed^43^,^44^, PAS and CAS are unique in that they can be defined for GO term pairs from different categories, e.g. for a term in Molecular Function (MF) and another one from Biological Process (BP). *CAS* and PAS are general-purpose scores for quantifying coherence of GO term pairs. The new contribution of this work is the development of GO-MEP, which combines multiple features including CAS and PAS, and that we showed that CAS and PAS is effective in improving accuracy of identifying missing genes. This study of GO-MEP showed that adding CAS and PAS substantially improved the accuracy of identifying missing genes in the yeast enzyme-enzyme network compared to the cases when only the conventional features, gene expression, phylogenetic profile, were used. The accuracy of GO-MEP was further boosted when function similarity between candidate genes to the missing enzyme was taken into account, even in the cases that the exact function of candidates are not revealed. Finally, it was also demonstrated that the accuracy improves by considering indirect neighbors to the target enzyme position in the network with a proper network-topology-based weighting scheme used.

## Results

We constructed an Enzyme-Enzyme Network (EEN) of *Saccharomyces cerevisiae* (yeast) (See Methods). The EEN contains 688 known enzymes with 5185 edges. GO-MEP uses six different scores, gene expression correlation (*EXPR*), phylogenetic profile (*PHYL*), *CAS*, *PAS*, *funsim*, and *PROFILE*, either individually or in combination of two or more scores to evaluate fitness of candidate genes to a target enzyme in the EEN. For a target enzyme position in the EEN, a prediction is considered as correct if the correct gene is ranked the top by the score among all the candidates. The prediction performance of GO-MEP with a score is evaluated by the score rank of the correct gene among all the candidates for a target enzyme.

First, we examined performance of individual scores from different angles. Then, we discussed prediction accuracy of GO-MEP using combination of scores. We further tested GO-MEP in a more difficult situation when genes for 20% of the nodes among the 688 nodes in the EEN are missing and needed to be filled.

### Network distance dependency of the scores

**Figure 1:**
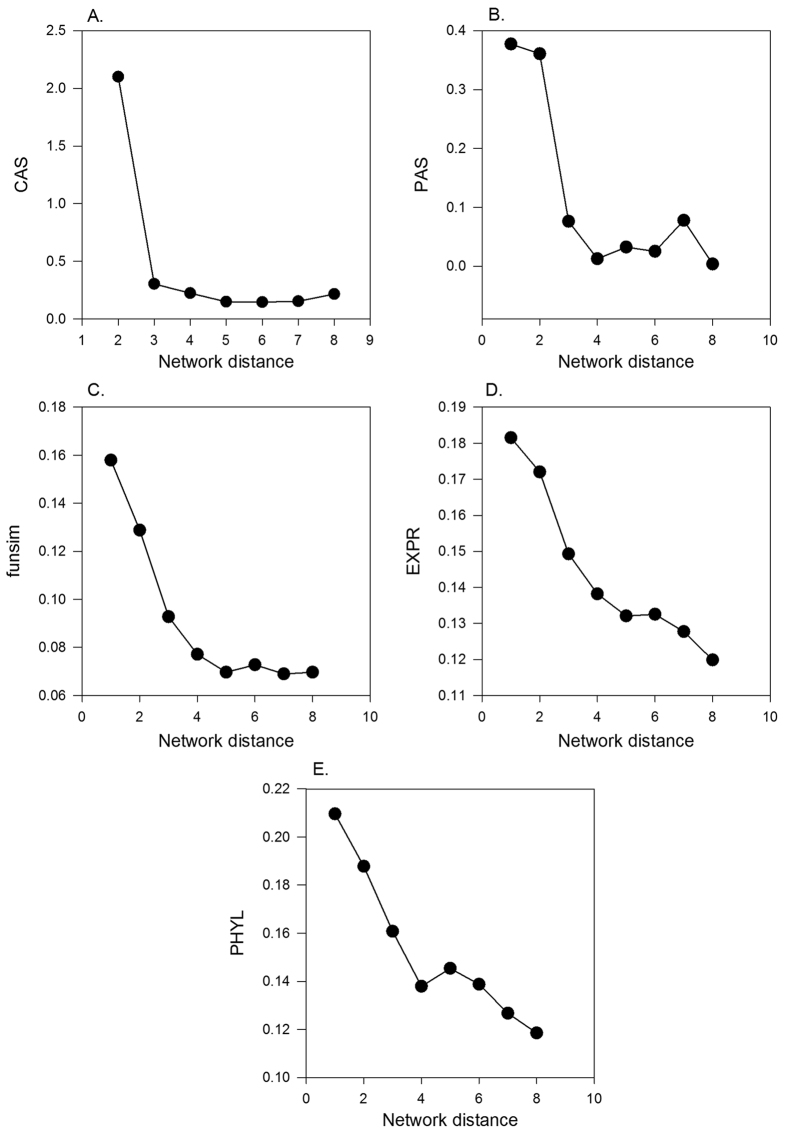
Correlation between the feature scores and the network distance. The average of feature scores relative to the network distance is plotted. (**A**) *CAS*; (**B**) *PAS*; (**C**) *funsim*; (**D**) *EXPR*; and (**E**) *PHYL*.

To begin with, we examined how each individual score changes relative to the networks distance. In Fig. 1, average of five scores, *CAS*, *PAS*, *funsim*, *EXPR*, *PHYL* computed for two nodes at different distances in the EEN are shown. All the scores showed a significant drop when the distance of the two nodes increased from 1 to 2 and from 2 to 3. At the network distance of 3 or more, *CAS* and *PAS* did not change much, whereas *funsim*, *EXPR*, and *PHYL* further showed gradual descent of the average scores as the network distance increases. The results of *EXPR* and *PHYL* are consistent with a previous work^25^.

**Figure 2:**
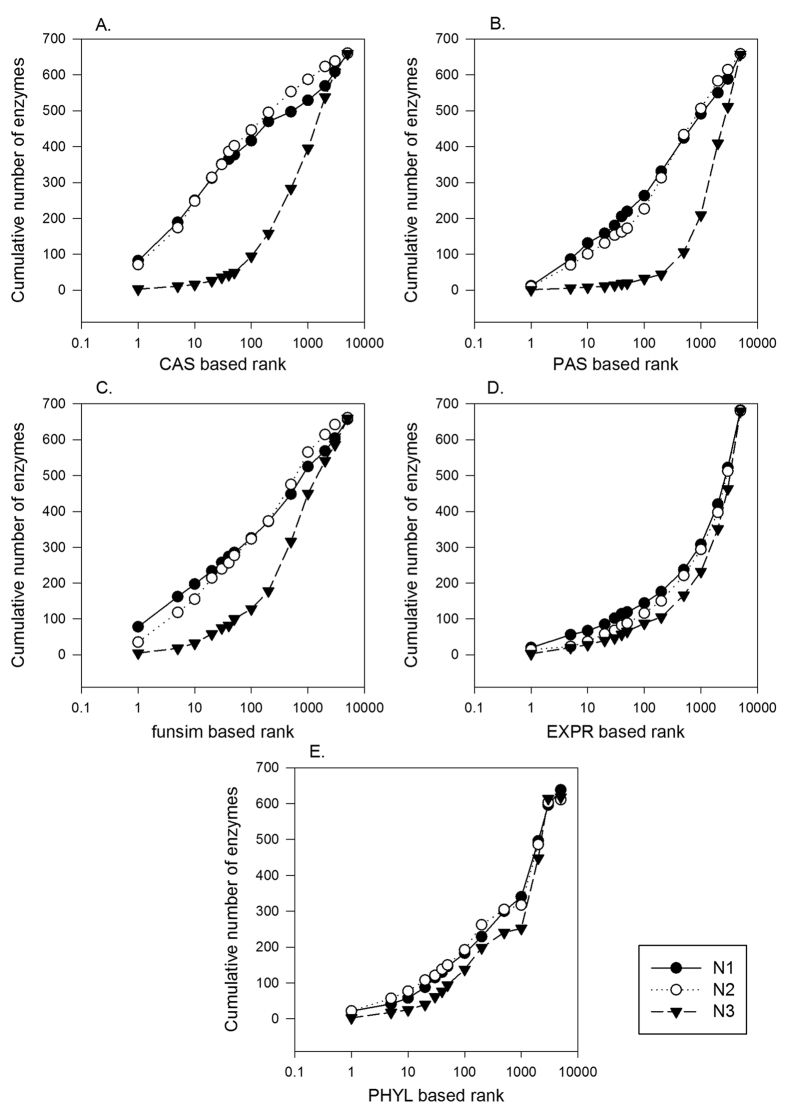
Enzyme assignment performance of the feature scores using different network neighbor levels. For each of the 688 enzyme positions in the EEN, five individual feature scores were used to rank the correct enzyme together with the 5199 negative proteins were ranked. Then, the cumulative number of enzymes (y-axis) having ranks better than a value on the x-axis was plotted. The score of a candidate protein for a target enzyme position was computed according to Eq. 7, with a consideration of the first (N1), second (N2) and third (N3) level neighbors (k = 1, 2, 3 in Eq. 7). (**A**) *CAS*. Mean Reciprocal Rank (MRR) of N1, N2, and N3 results were 0.199, 0.186, and 0.014, respectively. (**B**) *PAS.* MRR of N1, N2, and N3 were 0.072, 0.059, and 0.007, respectively. (**C**) *funsim*. MRR of N1, N2, and N3 were 0.173, 0.114, and 0.021, respectively. (**D**) *EXPR*. MRR of N1, N2, and N3 were 0.055, 0.06, and 0.020, respectively. (**E**) *PHYL*. MRR of N1, N2, and N3 were 0.054, 0.061, and 0.020, respectively.

In Fig. 2, we tested the individual scores for correct gene recognition at each of 688 enzyme position in the EEN. Given an enzyme position under consideration, the relatedness score (Eq. 7) was computed with an individual score for 1^st^, 2^nd^, and 3^rd^ level neighbors (*i.e.* k = 1, 2, 3) for 5200 candidate proteins (1 correct enzyme, and 5199 negative proteins). Then, the cumulative number of times that the correct enzyme was ranked within a certain top rank in terms of the relatedness score was reported in Fig. 2. In the best case, the feature score will be able to top rank the correct enzyme at the every position, and in the worst case the enzyme is ranked 5200^th^.

It is shown that for all the feature scores, the scores using the first (*N1*) and the second (*N2*) level neighbors showed comparable performance whereas the performance drops significantly when ranking was computed on the score using the third level (*N3*) neighbors. The performance difference between the first, second, against the third level scores were large for the GO annotation based scores, CAS, PAS, and *funsim*. Comparing the five feature scores with the first level network neighbors, CAS performed best considering the cumulative correct enzyme assignment within top 100 ranks. CAS ranked 416 enzymes correctly out of 688 target enzymes within top 100 and *PAS, funsim, PHYL,* and *EXPR* follow in this order with 263, 226, 183, and 143 correct assignments, respectively. *CAS* was also the best in terms of the Mean Reciprocal Rank metric (Equation 9) when N1 level neighbors were considered. MRR for *N1* for each score type was: *CAS*, 0.199; *PAS*: 0.07, *funsim*: 0.173, *PHYL*: 0.054, and *EXPR*: 0.055, respectively.

### Effect of GTOM weights for the score performance

**Figure 3:**
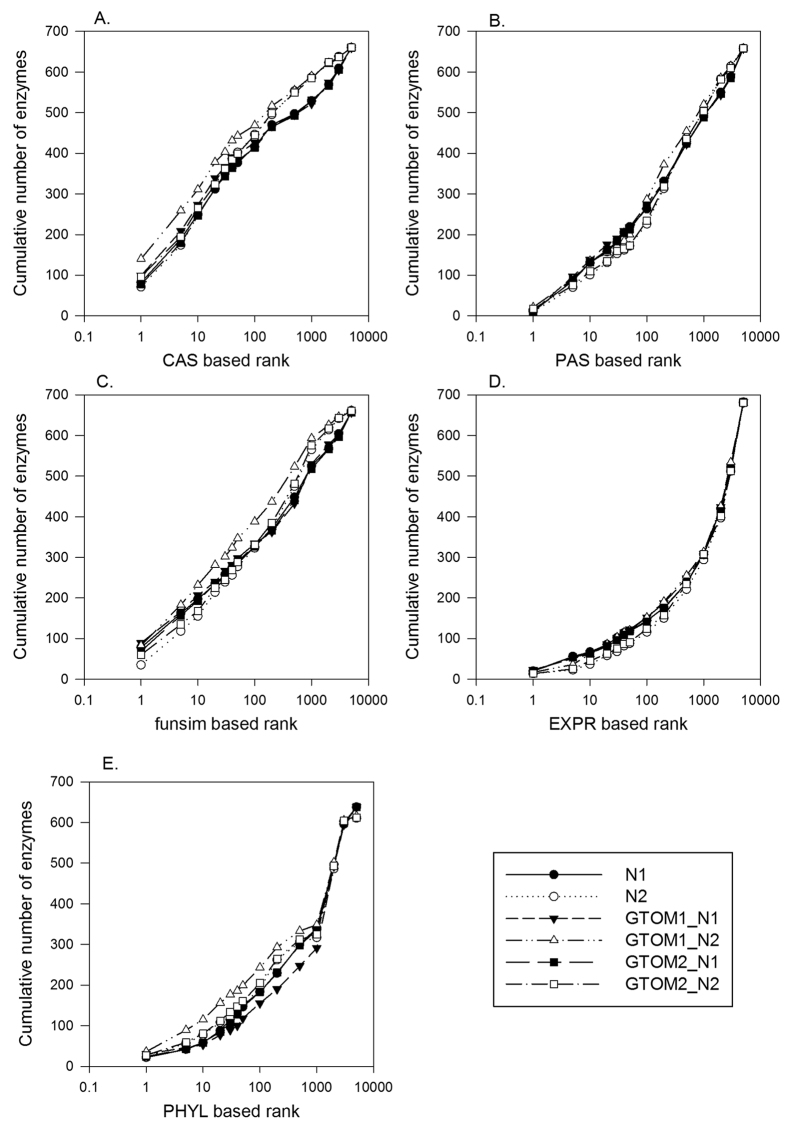
Score performance with multi-level neighbors and GTOM weights. For each feature score, six combinations of network neighbor levels and GTOM weights were compared in terms of the cumulative ranks of correct enzyme among 5200 candidates. The six variations of a score of type *t* are two neighbor levels from Eq. 7, and four combinations of two neighbor levels of the score, and two neighbor levels for GTOM weights. (**A**) *CAS*. MRR for *N1*, *N2*, *GTOM1_N1*, *GTOM1_N2*, *GTOM2_N1*, and *GTOM2_N2* were 0.199, 0.186, 0.223, 0.294, 0.192, and 0.219, respectively. (**B**) *PAS*. MRR were 0.072, 0.059, 0.077, 0.083, 0.072, and 0.070, for the same order of the score forms as *CAS*. (**C**) *funsim*. MRR values were 0.173, 0.114, 0.186, 0.191, 0.164, and 0.143, respectively. (**D**) *EXPR*. MRR were 0.055, 0.032, 0.055, 0.046, 0.053, and 0.034, respectively. (**E**) *PHYL*. MRR were 0.054, 0.061, 0.060, 0.096, 0.056, and 0.069, respectively.

Next, we examined the performance of individual scores with GTOM weights with two network neighbor levels. As described in Methods, GTOM quantifies a topological distance between two nodes in a network (Equation 6). Among neighboring nodes at the same network distance (e.g. 2) to a target enzyme position in the EEN, nodes have higher GTOM weights than others if they share more common connected nodes between the target. For each score type, prediction performance of six score forms that come from combinations of two network neighbor levels (N1 and N2) and three GTOM weights (no weight as shown in Eq. 7, GTOM1 and GTOM2 as shown in Eq. 8) was examined in Fig. 3. MRR of all the score variations are shown in the figure caption. In terms of MRR, the *GTOM1_N1* and *GTOM1_N2* options showed a better or the same performance as the non-weighted options (i.e. N1 and N2) for all the score types but one (*PHYL* for *GTOM1_N1* and *EXPR* for *GTOM1_N2*), although often the improvement was marginal. For *CAS*, *funsim*, and *PHYL*, *GTOM1_N2* (triangles in the graphs) performed best with a relatively large margin to the other score variations.

### Performance comparison with different GO annotation levels

Levels of function annotation in the current database varies from gene to gene with a substantial fraction of genes under-annotated with less specific GO terms or no annotation due to lack of experimental evidence. There are also cases that annotations are based on computational predictions with rather general GO terms, *e.g.* transporter, kinase, etc. In this section, to mimic the situation where complete annotation information is not available for candidate proteins, we examined how the accuracy of the enzyme assignment changes by using different lower levels of GO annotations.

We used five levels of candidate protein GO annotations for enzyme recognition for the EEN and tested the four GO term-based scores, i.e *CAS*, *PAS*, *funsim*, and *PROFILE*. *PROFILE* directly computes similarity of a target enzyme function that are converted from its Enzyme Commission (EC) number to GO annotations of candidate genes (See Methods). Annotation levels included all GO annotations in the database and parental terms mapped from the original GO annotations to level (depth) 3, 4, 5, and 6 in the GO hierarchy. In the parental term mapping, terms that are at a deeper level than the target level were mapped to their parental terms at the target level, but those that are at a shallower level are kept intact. The level of a GO annotation term is defined as its maximum distance from the Gene Ontology root node “all”. For example, using a GO term in the MF category, “MAP kinase tyrosine phosphatase activity (GO:0033550)”, is located at the 9^th^ level in the hierarchy; its 6^th^ level parental term is “phosphatase activity (GO:0016791)”. Its 5^th^, 4^th^, and 3^rd^ parental terms are “phosphoric ester hydrolase activity (GO:0042578)”, “hydrolase activity acting on ester bonds (GO:0016788)”, “hydrolase activity (GO:0016787)”, respectively.

**Figure 4:**
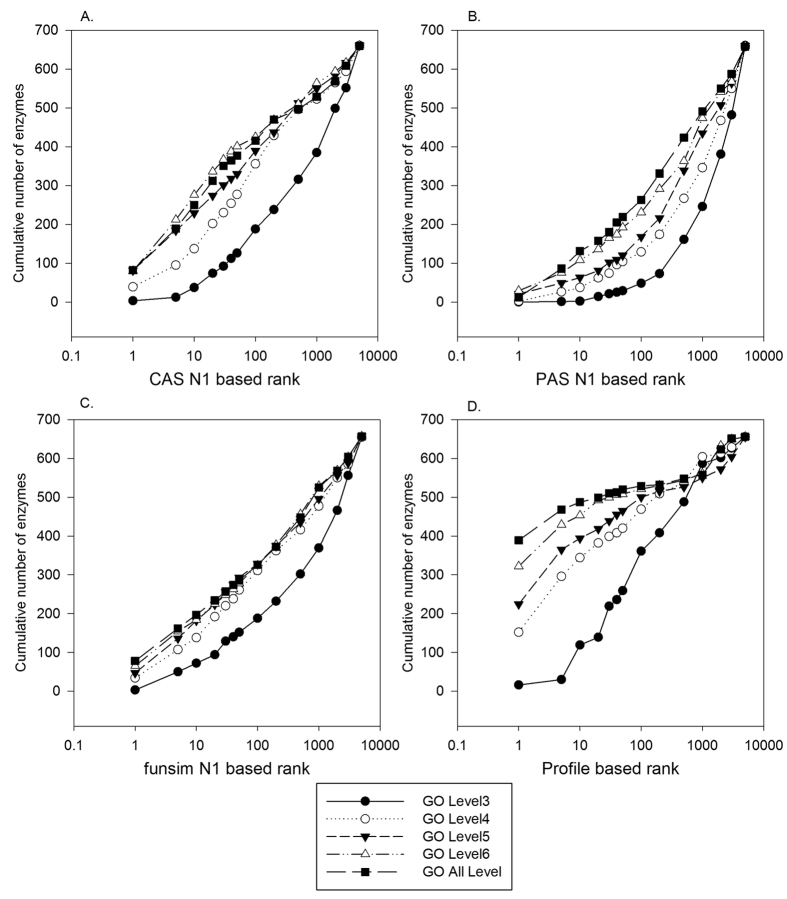
Performance of GO-based Feature scores with different GO levels. The performance of *CAS*, *PAS*, *funsim*, and *PROFILE* (the cumulative number of correct enzyme recognized within certain top ranks) was examined using five different resolution levels of GO terms of candidates, parental term mappings to the 3^rd^, 4^th^, 5^th^, and 6^th^ level in the GO hierarchy as well as the original GO annotations. The relatedness score (Eq. 7) with the 1^st^ level neighbors (*k* = 1) was used (showed *N1* in the figure legend). (**A**) *CAS*. MRR using the GO level 3, 4, 5, 6, and the original annotation were 0.002, 0.104, 0.184, 0.207, and 0.199, respectively. (**B**) *PAS*. MRR were 0.004, 0.023, 0.054, 0.081, and 0.072, respectively, for the same order of GO annotation levels as *CAS*. (**C**) *funsim*. MRR were 0.041, 0.104, 0.135, 0.155, and 0.173, respectively. (**D**) *PROFILE*. MRR were 0.058, 0.314, 0.416, 0.541, and 0.617, respectively.

For *CAS* (Fig. 4A), interestingly, prediction performance did not deteriorate much with the parental terms up to the 5^th^ level. Using the 4^th^ level terms (empty circles), early rank recognition of the correct enzyme, *e.g.* within the top 10 ranks, declined to about half, however, the difference was smaller when top 100 ranks were considered. Reflecting the deterioration of the early rank recognition using the 4^th^ level terms, MRR using the 4^th^ level terms (0.104) showed a 56.5% drop from that of the 5^th^ level terms (0.184), which is larger than the drop from 6^th^ level (0.207) to 5^th^ level (88.9%). When parental terms on the 3^rd^ level were used, the recognition worsened largely even at lower rank cutoffs. MRR for the 3^rd^ level was 0.021, 20.2% of that of the 4^th^ level.

The accuracy by *PAS* (Fig. 4B) was more affected by lowering the resolution of GO terms than *CAS*. In the case of *funsim* (Fig. 4C), accuracy did not change much up to the 4^th^ GO level and a substantial decline of the accuracy started from the 3^rd^ level. *PROFILE* showed a different trend from the other three scores (Fig. 4D). Lowering the resolution of GO terms affected to the accuracy more sensitively than the other scores for earlier rank recognition. The number of EEN positions that ranked the correct enzyme at the top was 389 when all the annotated GO terms were used, which decreased almost evenly to 322, 224, and 152 using GO terms mapped to 6^th^, 5^th^, and 4^th^ levels, and larger to 16 using 3^rd^ levels, respectively. This is consistent when MRR was considered. MRR changed almost evenly from 0.617 using all the GO annotations to 0.541, 0.416, and 0.314 using the 6^th^, 5^th^, and 4^th^ level GO terms, and dropped largely to 0.058 using the 3^rd^ level GO terms. The deterioration of the performance by lowering GO levels was observed at early recognition. When the top 100 ranks were considered, the accuracy up to the GO term level 4 showed similar performance.

To summarize, for all four scores, low resolution GO terms at the 3^rd^ level substantially deteriorated the accuracy of identifying correct enzymes. Deterioration of the performance by lowering GO resolution was observed mainly at early recognition within the top 100 ranks. While *PROFILE* showed the most sensitive decline of accuracy as lower GO term resolutions were used, the other three scores showed stable performance to the parental term mapping at up to the 5^th^ level.

### Missing enzyme identification with different feature scores

**Figure 5:**
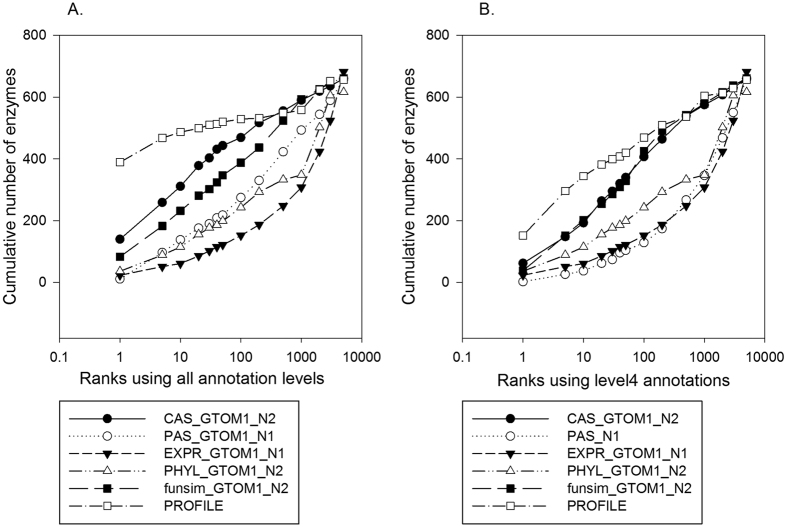
Performance comparison of individual feature scores. Best performing form among six variations (Eqns 7 and 8) from *CAS*, *PAS*, *EXPR*, *PHYL*, *funsim* as well as *PROFILE* were compared in terms of ranks of correct enzymes at each EEN position. *N1* and *N2* in the figure legend stand for *k* = 1 and 2 for Rel_Score. (**A**) Feature scores computed using the original GO annotations. MRR for *CAS*, *PAS*, *EXPR*, *PHYL*, *funsim*, and *PROFILE* were 0.294, 0.077, 0.055, 0.096, 0.191, and 0.617, respectively. (**B**) Scores computed using parental GO terms mapped at the 4^th^ level. MRR for *CAS*, *PAS*, *EXPR*, *PHYL*, *funsim*, and *PROFILE* were 0.156, 0.024, 0.055, 0.096, 0.134, and 0.314, respectively.

Up to the previous section, we examined individual score types in different settings. Here, we directly compared the performance of six individual feature scores in the missing enzyme recognition. For *a* score type, the best score form among the six variations (Eqns 7 and 8) was used, which gave the largest cumulative number of correct enzymes as the rank 100. In Fig. 5, two results are shown: the left panel shows the results when all the original GO annotations were used for the candidate proteins, while in the right panel a GO term was mapped to its parental term at the 4^th^ level in the GO hierarchy. As were done in the previous sections (Figs 2, 3 and 4), for each of the 688 positions in the EEN, the correct enzyme together with 5199 other proteins were ranked in terms of the score, and the rank of the correct enzyme was reported.

When original GO annotations were used (Fig. 5A), *PROFILE* significantly outperformed the other scores. In particular, when rank 1 correct enzyme recognition was considered, the cumulative number of correctly recognized enzymes was more than twice larger (389) than the other scores. MRR gives a consistent view. MRR of *PROFILE* is 0.617, which is 2.1 times larger than that of *CAS* (0.294), the second best performing score. *funsim* and *PAS* follow to *CAS* in this order, thus the four GO-term based scores showed better performance than *PHYL* and *EXPR* in terms of the correct enzyme ranking and MRR. *EXPR* had the least relevant information for identifying missing enzymes.

When only low resolution GO terms at the 4^th^ level or lower are available (Fig. 5B), *PROFILE* still showed the best performance although the number of correctly recognized enzymes at rank 1 and MRR dropped to about half (152 and 0.314, respectively). Note that the performance of *PHYL* and *EXPR* are the same between Fig. 5A,B because they are not relevant to GO annotations. *CAS* and *funsim* now showed almost the same performance, which was still better than *PHYL.* Interestingly, *PAS* was severely affected by lower resolution GO terms and its performance became worse than *EXPR*. *PAS*’s MRR became less than half (0.024) from *EXPR*’s (0.055).

### Predicting missing enzymes using and score combinations

Finally, we combined the feature scores using L2 normalized logistic regression classifier and examined its performance. We built classifiers with three different feature combinations. The first one combines *EXPR* and *PHYL*, the two features used in previous works 22,25,31. The second combination is with *CAS, PAS, EXPR, PHYL,* and *funsim*, and *PROFILE* was added to those as the third combination. Similar to Fig. 5, we used two GO term settings, one with all the annotated GO terms and lower resolution mapping to the 4^th^ level.

**Figure 6:**
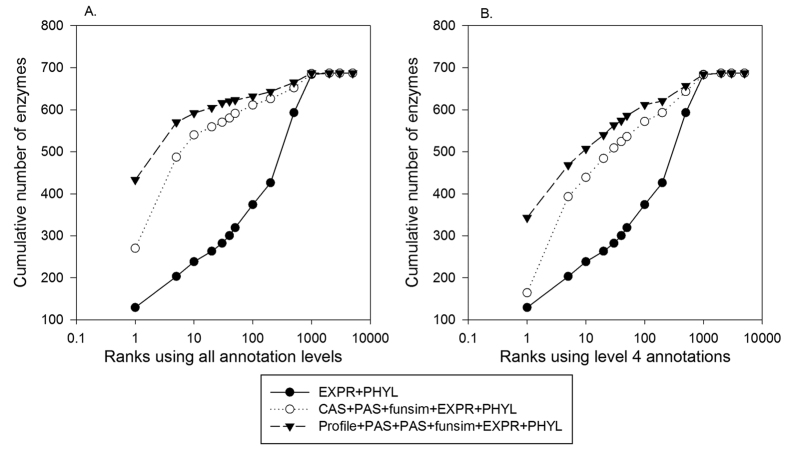
Missing enzyme prediction by combining feature scores. The six feature scores were combined in three different ways, *EXPR*+*PHYL*, *CAS*+*PAS*+*funsim*+*EXPR*+*PHYL*, and *Profile*+*CAS*+*PAS*+*funsim*+*EXPR*+*PHYL*, in the framework of L2 normalized logistic regression. The combinations were used for ranking correct enzyme along with 5199 negative proteins for each of the 688 enzyme positions in the EEN. For all the feature scores except for *PROFILE*, two forms of the scores, *GTOM1_Rel_Score*^*1*^ and *GTOM1_Rel_Score*^*2*^ were used. (**A**) Feature scores computed using the original GO annotations. MRR were 0.244, 0.535, and 0.723 for the *EXPR*+*PHYL*, *CAS*+*PAS*+*funsim*+*EXPR*+*PHYL*, and *Profile*+*CAS*+*PAS*+*funsim*+*EXPR*+*PHYL* combinations, respectively. (**B**) Scores computed using parental GO terms mapped at the 4^th^ level. MRR were 0.241, 0.386, and 0.583 for the score combinations in the same order.

In the both GO term settings, neighboring protein information by adding GO-based features, *CAS*, *PAS*, and *funsim*, made substantial improvement over *EXPR*+*PHYL* in selecting correct enzymes for enzyme positions in the EEN. The combination of five feature scores ranked 611 enzyme positions correctly within top 100 as compared to 374 by the combination of *EXPR* and *PHYL*. In terms of MRR, the five score combination improved MRR to 0.535 from 0.244 by *EXPR* and *PHYL*. Apparently, the result by the five feature score combination is a significant improvement over the performance when the five scores was used individually (Fig. 5A). Although the *EXPR* and *PHYL* combination fell behind, the combination’s performance is still a large improvement over the individual scores, which ranked 144 and 183 enzyme positions correctly within the top 100 ranks when they are used individually (Fig. 5A). MRR of *EXPR* and *PHYL* only were 0.055 and 0.096 in Fig. 5A, which was increased to 0.244 by the combination of the two. Adding the PROFILE score further improved the performance. Compared to the *PROFILE*-only result, MRR by the six score combination with *PROFILE* increased by 17.2% from 0.617 to 0.723 when the original GO annotations were used (Figs 5A and 6A). When the level 4 GO annotations were used (Figs 5B and 6B), the performance improvement in terms of MRR by the six score combination over the *PROFILE*-only is further increased to 85.7% from an MRR of 0.314 (Fig. 5B) to 0.583 (Fig. 6B). This may be probably because the *PROFILE*’s sensitivity to GO annotation levels observed in Fig. 5 was compensated by the other scores that are less sensitive to lower GO annotation levels.

Lastly in this section, we discuss GO-MEP’s results in comparison with earlier works which performed missing enzyme finding for a yeast enzyme network. Note that a completely fair comparison is not possible because each work used different testing data and feature combinations, even in cases that same type of features were used. Kharchenko *et al*.^22^ used gene expression profiles of neighboring genes of a target enzyme to find missing enzyme in an yeast EEN. Out of 564 metabolic enzymes, they identified 23 (4.08%) at top rank and approximately 23% within top 50 ranks. Similarly, Chen *et al*.^25^ used a phylogenetic profile and ranked 50% enzymes within the top 100 ranks in a leave one out analysis. Another work by Kharchenko *et al*.^31^ combined information from phylogenetic profile, expression profile, gene fusion, and chromosome clustering to predict missing enzymes in the yeast EEN and showed almost 50% of the enzymes were ranked within top 10 ranks. Compared to these methods, GO-MEP with the *Profile*+*CAS*+*PAS*+*funsim*+*EXPR*+*PHYL* combination ranked the correct gene at the top rank for 49.9% (343 out of 688) of the enzyme positions and 73.7% (507 out of 688) within top 10 ranks using level 4 GO annotations (Fig. 6B). The comparison indicates that GO-MEP performs better than the exiting methods and that GO-based features are effective to identify missing genes.

### Filling multiple missing enzymes in the network

**Figure 7:**
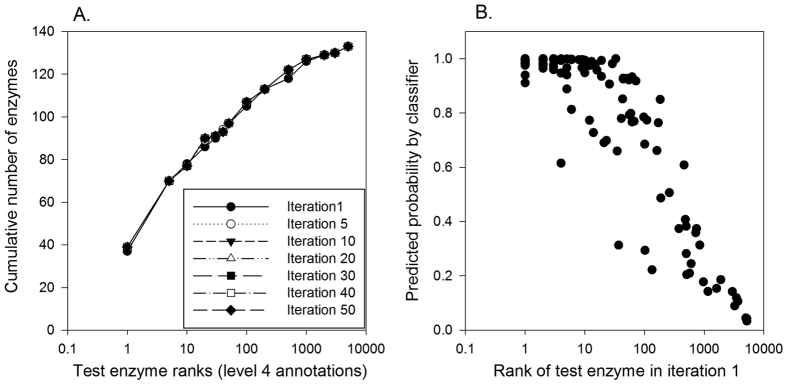
Performance on predicting multiple missing genes. Among the 688 nodes in the EEN, genes for 20% (*i.e.* 137 nodes) are predicted using GO-MEP trained on the rest of 80% (551 nodes). For each node used for training, one correct enzyme and 1000 negative proteins were used. Five feature scores, all except for *PROFILE* were used. (**A**) The number of correct enzymes ranked within certain ranks were reported at iterations 1, 5, 10, 20, 30, 40, and 50. In each iteration, proteins with a probability of 0.9 or above were assigned. MRR for each iteration was 0.378, 0.387, 0.382, 0.382, 0.382, 0.378, and 0.377 at the 1st, 5th, 10th, 20th, 30th, 40th, and 50^th^ iteration, respectively. (**B**) Assigned probability of correct enzymes at the 137 nodes relative to their ranking in the first iteration.

We further tested GO-MEP in a more difficult situation when genes for 20% of the nodes (137 nodes) among the 688 nodes in the EEN are missing. L2 regularized logistic regression with five features scores, *CAS, PAS, EXPR, PHYL*, and *funsim*, were trained on the rest of the 80% of the nodes (*i.e.* 551 nodes), each of which have the correct enzyme and 1000 negative proteins. Mapped GO terms at the 4^th^ level were used. *PROFILE* was not included in the score because it directly evaluates compatibility between a missing enzyme position and candidate genes and thus does not depends on gene assignment of neighboring nodes. After the training, proteins were assigned to missing enzyme positions in the network in an iterative fashion. As performed in the previous sections, the correct gene was included among the other 5199 negative proteins. At the beginning, a random candidate was assigned to each missing node, while in the subsequent iterations (Iteration 1 and the following iterations in Fig. 7A) a candidate protein that has the highest probability to a node was assigned to the node, if the probability is larger than a cutoff, 0.99. In the second and later iteration, proteins assigned to neighboring nodes in the previous iteration contribute in providing functional coherence scores (*i.e. CAS*, *PAS*, and *funsim*) for a missing enzyme position. The iterative process was repeated for 50 times.

**Figure 8:**
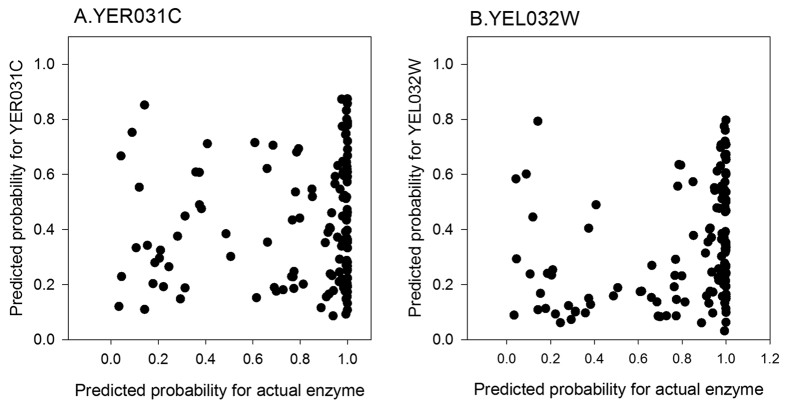
Probability comparison between the actual enzymes and other proteins. Using (**A**) YER031C and (**B**) YEL032W as examples, probability of the actual enzymes of the 137 positions in the EEN and that of the example protein are compared. Probability computed for the first iteration of the computation was used.

Figure 7A shows the performance of GO-MEP at selected iterations. Overall it turned out that there was not much change in the performance over the iterative process. MRR computed at the iteration 1, 5, 10, 20, 30, 40, and 50 are very close to each other ranging between 0.377 to 0.387. MRR increased from 0.378 at the iteration 1 to 0.387 at iteration 5, but it then slightly deteriorated in the later iterations. Figure 7B shows that correlation between the rank of the correct enzyme and the probability assigned for the correct enzyme for 137 test nodes. Notably, the probability and rank of the correct enzyme at test nodes correlate well, indicating that assignment with a high probability, *e.g.* 0.9, has a high chance that the predicted protein at the top rank is correct, or if not, that the correct enzyme is included within proteins with the top ~10 ranks. In Fig. 8, using two example genes, YER031C and YEL032W, we examined the probability assigned to the correct enzyme for the 137 test nodes relative to the probability computed for each of the example genes. As shown in Fig. 8A,B, for most of the test nodes the actual enzyme has a higher probability, which is closer to 1.0 whereas the two example genes have a probability of less than 0.9 for almost all the cases.

To summarize this section, Fig. 7A shows that in an extreme case where as large as 20% of genes are missing in the network, GO-MEP is able to identify missing enzymes among top ranks of candidates, *e.g.* within top 10 ranks for more than half of the cases. Moreover, it is shown in Figs 7B and 8 that the probability assigned to candidates can accurately indicate the likelihood that the prediction is correct, because the rank of the correct enzyme and the probability correlates well.

## Conclusion

Pathway reconstruction for an organism is an effective way for elucidating characteristics of the organism and also a crucial step to lead to quantitative pathway simulations. A practical challenge in the reconstruction is that not all the enzyme genes can be easily found due to lack of significant sequence similarity to known genes for target enzymes. In previous works, other features of genes, including gene expression profiles, phylogenetic profiles, and comparative genomics features were used to find similarity between candidate genes and known enzymes that are in the neighborhood to the target enzyme in the EEN. The contributions of the current work is three fold: First, we have developed a missing gene prediction method, GO-MEP, which showed substantially better performance than previous works. Particularly, we demonstrated that GO-based scores including CAS and PAS are effective for identifying missing genes. Second, we introduced the GTOM weights to the missing gene finding problem and showed that the weights shows some improvement in accuracy of individual feature scores. Third, in addition to the test to fill one node at a time as performed in previous works, we demonstrated that GO-MEP can also identify missing genes even in the case that 20% of the genes are missing in the EEN. Moreover, the probability computed for candidate genes correlates well to the accuracy of the assignment.

The current work was limited in its scope in identifying missing enzyme genes in the EEN. However, the developed method, GO-MEP, could be applied to more general missing gene finding problems in other biological contexts, such as finding genes in transport systems or host cell invasions. Extending the method to such a general framework is left as a future work.

## Methods

### Enzyme-Enzyme Network (EEN)

We constructed an Enzyme-Enzyme Network (EEN) of *Saccharomyces cerevisiae* (yeast) in a similar way as in a previous work^31^. In the EEN each node represents an enzyme and each edge represents that the connected enzymes share metabolites in the reactions they catalyze. From the BiGG database^45^ 1149 validated enzymatic reactions of *Saccharomyces cerevisiae* were obtained. Out of the 1149 reactions, 810 were associated with 750 enzyme proteins while 339 reactions are unassigned to any protein. Enzymes are connected by an edge if they share a common metabolite in their reactions unless the metabolite is among abundant metabolites, which consist of ATP, H2O, AMP, ADP, CO2, NH4, NAD(H), NADP(H), CoA, glutamate-L, phosphate, diphosphate, and hydrogen, because these metabolites produce non-functional specific connections if considered^22^,^31^. When there are more than one enzymes associated with a single reaction then all of them are connected in the EEN. When a reaction does not have any ORF associated with it, the reaction was associated with a pseudo enzyme with the reaction identifier and used in the EEN connections based on its shared metabolites. The resulting network had 1009 nodes (more than the number of known enzymes since pseudo enzymes were considered) and 8362 edges. In the study below, prediction accuracy was measured for known 688 enzymes with 5185 edges that are connected to the EEN.

### New missing enzyme prediction method, GO-MEP

We developed a new method named GO-MEP (GO-based Missing Enzyme Predictor) for finding missing genes that integrates multiple features, gene expression, phylogenetic profile, two function association scores, Co-occurrence Association Score (*CAS*) and PubMed Association Score (*PAS*)^42^, and two GO similarity scores, *funsim* and *PROFILE*. Below we first explain each scoring feature, and then weights assigned to features that reflect the distance on the network topology (GTOM), and finally how the weighted features are combined in GO-MEP.

### Phylogenetic profile correlation score

To identify missing genes in the network, six different scores were considered, which describe different biological contexts of candidate genes. The first score is a phylogenetic profile. A phylogenetic profile of a protein indicates existence or absence of its homologs in genome sequences, which is represented as a vector of ones and zeros. Orthologs of a protein in the EEN was identified by running BLAST^46^ with an E-value cutoff of 10^−3^ against a collection of 70 evolutionarily dissimilar prokaryotic and eukaryotic genomes^47^. The genome sequence files were obtained from the KEGG database^4^. For two proteins similarity of phylogenetic profile was quantified with the Pearson’s correlation (1.0, the highest similarity, −1.0, the least correlation).

### Expression profile correlation score

For each protein in the EEN an expression profile was created using the Rosetta’s compendium reference dataset^48^. The dataset is based on cellular perturbations across 300 diverse mutations and chemical treatments. For a pair of proteins, the absolute value of Spearman’s rank correlation was used to quantify correlation of their expression patterns.

### *CAS* and *PAS* association scores

Three Gene Ontology (GO)-based function scores were used as protein functional features, Co-occurrence Association Score (*CAS*), PubMed Association Score (*PAS*)^42^, and the *funsim* score^33^,^49^. GO annotations for yeast enzymes are obtained from the GOA database^50^. Inferred Electronic Annotations (IEA) were excluded from GO annotations for better reliability. *CAS* and *PAS* are designed for quantifying protein function coherence rather than simple functional similarity so that they can identify proteins involved in the same biological context^42^. For a pair of GO terms *CAS* is defined as the ratio of the frequency that both GO terms are used to annotate single gene relative to the frequencies of the individual GO terms to annotate the genes independently.

Similarly, *PAS* for a pair of GO terms has been defined as the ratio of number of abstracts in which the GO term names co-occur as opposed to the number of times the individual GO term names occur independently in the abstracts. More concretely, we used the NCBI’s Entrez ESearch utility for obtaining the count of PubMed abstracts related to the particular GO terms. For example, for computing the PubMed association between terms *GO:0003700* and *GO:0051169*, we first obtain their respective term names as *‘transcription factor activity’* and *‘nuclear transport’* from the GO database and remove words ‘and, or, not’ from their GO term names. The remaining words in the name are used to construct URL, *e.g*. http://eutils.ncbi.nlm.nih.gov/entrez/eutils/esearch.fcgi?db=pubmed&retmode=xml&rettype=full&term=transcription+factor+activity, which yields an xml that is then parsed to obtain the count of PubMed abstracts associated with the given term. For retrieving the counts of abstracts with two GO terms we appended the terms in the query URL and obtain the count. The search counts any abstract that simply mentions all the words that are appended in the URL. The ESearch query interface uses the MeSH indexing to incorporate the synonyms and the term variations. This provides us with a convenient way to retrieve the information that has been represented using different terms for the same concepts. The January 2010 version of the PubMed database was used.

For pair of proteins *X* and *Y*, each of which have multiple GO term annotations, *CAS* and *PAS* scores are computed as shown in Eqns 1 and 2, respectively, by averaging the pairwise *CAS* and *PAS* scores between their GO annotations:


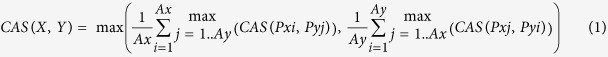



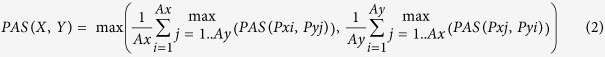


here *A*_*x*_ and *A*_*y*_ are the number of GO annotations for proteins *X* and *Y* respectively, and *P*_*xi*_ is *i*^*th*^ annotation for protein *X* and *P*_*yj*_ is *j*^*th*^ annotation for protein *Y*.

### *funsim* similarity score

The *funsim* score^49^ was used as an additional function-based score to compute similarity between candidate protein and its neighbors in the EEN. Consider GO terms *c1* and *c2* whose similarity is computed as Eq. 3 where *c* represents their common ancestor and *p(c)* is defined as the fraction of proteins in GOA database annotated with GO term *c*.


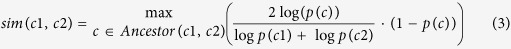



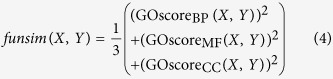



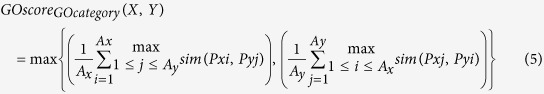


For two proteins *X* and *Y, funsim(X, Y)* is defined as Eq. 5 where *GOscore*_*category*_ is the similarity between GO annotations of *X* and *Y* for a particular GO category BP, MF or CC. *GOscore*_*category*_ is computed by averaging the *sim* scores between GO annotations of *X* and *Y* in the given category as presented in Eq. 5, where *A*_*x*_ and *A*_*y*_ are the number of annotations for proteins *X* and *Y* respectively in that category, and *P*_*xi*_ is *i*^*th*^ annotation for protein *X* and *P*_*yj*_ is *j*^*th*^ annotation for protein *Y*.

The *funsim* score was also used to directly compare GO terms of candidate proteins against GO term profile of the target enzyme (*PROFILE*), which were mapped from its enzyme commission (EC) number^51^ and subcellular localization information. The GO term mapping of the Molecular Function (MF) and Biological Process (BP) category was obtained by an automatic mapping provided by the Gene Ontology website (www.geneontology.org). BP terms were also often manually mapped from the subsystem information in the BiGG database. Cellular Component (CC) terms were mapped from the localization information of genes provided in the BiGG database, which classifies the localization into eight locations (cytosol, extracellular, Golgi apparatus, mitochondria, nucleus, endoplasmic reticulum, vacuole and peroxisome). The *funsim* score between the target and a candidate genes is called the *PROFILE* score.

### Generalized Topological Overlap Measure (*GTOM*)

For a target missing enzyme position in the EEN, a candidate gene is evaluated considering feature correlation of the candidate against neighboring enzymes in the EEN. Among neighboring genes at the same distance to the target position, we can consider that some of them are more closely related than others to the target if they share more common nodes between the target. To capture this topological distance between a neighboring enzyme and the target, we used a weighting scheme, named Generalized Topological Overlap (GTOM) measure^52^. GTOM was designed to capture the functional relatedness between pairs of proteins in a network based on the number of shared interconnections between them. For a pair of proteins *X* and *Y*, *GTOMm* score is defined as


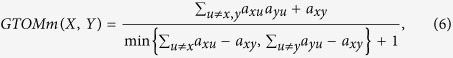


if *x* ≠ *y*, and 1 if *x* = *y*. *a* is the *m*^*th*^ level adjacency matrix for the network. In case of *GTOM1 (i.e. m* = 1), *a*_*xy*_ will represent the first level neighborhood for node *x* with 1 indicating an edge between two nodes *x* and *y* and 0 otherwise. For *GTOM2*, *a*_*xy*_ will be 1 if the node *x* is connected to node *y* within a path length of 2 and it will be 0 otherwise. Thus *GTOMm(X, Y)* measures the ratio of number of shared neighbors between *m* neighborhoods of two proteins *X* and *Y,* against the minimum degree (i.e. number of connections) among both the proteins. Here we have combined GTOM score between candidate and neighbors of a particular node in the EEN with the pairwise association scores between them.

### Combination of features in GO-MEP

To identify missing genes for an enzyme position in the EEN, six different scores discussed above were considered in GO-MEP, *i.e.* gene expression similarity (*EXPR*), correlation of phylogenetic profile (*PHYL*), and four function association scores, *i.e. CAS, PAS, funsim*, and *PROFILE*. These scores except for *PROFILE* were computed between a candidate gene to enzymes in the EEN that are 1^st^ and 2^nd^ level topological neighbors of the target enzyme. Below we explain how the score from each feature were combined in GO-MEP.

For a given enzyme position *i* in the EEN and a given candidate gene *j*, the score of type *t* that evaluates the relatedness of the candidate to the target enzyme, *Rel_Score*, is computed considering the k-th level neighbors as follows:


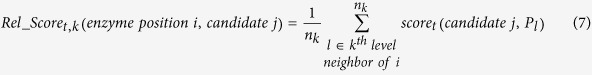


here *n*_*k*_ is the total count proteins at the *k*^*th*^ level neighbors to the enzyme *i*, *k* is either 1 or 2, *P*_*l*_ is a protein in the *k*^*th*^ level neighbors, and *score*_*t*_ is the score of type *t*, where *t* is one from *EXPR, PHYL, CAS, PAS,* or *funsim*. The score is further weighted by considering the GTOMm weight between the target enzyme in the EEN and its neighboring enzymes as follows:


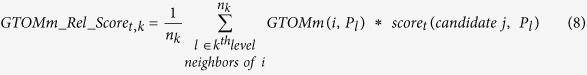


where *m* = 1, or 2 and *k* = 1, or 2. Thus, for *EXPR, PHYL*, *CAS, PAS,* and *funsim*, six combinations of scores with *m* and *k* (including without the GTOMm weighting, *i.e.* Eq. 7) were computed. For example, the gene expression similarity score, *EXPR*, *Rel_Score*_*EXPR,1*_, *Rel_Score*_*EXPR,2*_, which is based on Eq. 7 with the 1^st^ and 2^nd^ level neighbors to the target enzyme, as well as *GTOM1_Rel_Score*_*EXPR,1*_, *GTOM1_Rel_Score*_*EXPR,2*_, *GTOM2_Rel_Score*_*EXPR,1*_, and *GTOM2_Rel_Score*_*EXPR,2*_, which were computed based on Eq. 8. In addition, the PROFILE score was computed as the pairwise *funsim* score between GO functional profile constructed for enzyme position *i* in the EEN and the GO annotations of the candidate *j*.

These scores were combined using the L2 regularized logistic regression available in the LIBLINEAR package^53^. As discussed in the Results section, different combinations of scores were tested. For the results shown in Fig. 6, three different combinations of score types were used: (*EXPR* and *PHYL*), (*CAS*, *PAS*, *funsim*, *EXPR*, and *PHYL*), and (*PROFILE, CAS*, *PAS*, *funsim*, *EXPR*, and *PHYL*). For the results in Fig. 7, the five score type combination was used: *CAS*, *PAS*, *funsim*, *EXPR*, and *PHYL*. For each score type except for *PROFILE*, two forms of the scores, *GTOM1_Rel_Score*^*1*^ and *GTOM1_Rel_Score*^*2*^ (i.e. GTOM1 with two network neighbor levels, N1 and N2) were used because the performed well in Fig. 3. We conducted a leave one out analysis on the enzyme nodes in the EEN wherein when processing a particular enzyme position, we used positive and negative candidate examples from all the other 687 enzyme positions than the one under consideration. For training the classifier, negative proteins for a target enzyme were a sample of 1000 proteins out of 5199 yeast proteins excluding the 688 known enzymes was used. For countering bias of a higher number of negative examples in the training set, a weight of 0.001 was used for negative proteins. Since CAS and PAS has a large range of raw score values^42^, logarithmic conversion has been applied to the scores.

Performance of GO-MEP was measured in terms of the rank of the actual enzyme for the position based on the classification probability relative to the other 5199 negative proteins. In the results figures (Figs 2, 3, 4, 5, 6 and 7A), cumulative number of enzyme positions where the correct gene is selected within each score rank is reported. We also reported Mean Reciprocal Rank (MRR)^54^:


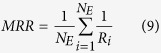


where *N*_*E*_ is the number of enzyme positions queried and *R*_*i*_is the rank of the correct gene among candidates for the i-th enzyme position in the ENN. If a prediction method always selects the correct gene at the top of the rank, MRR is 1.0, the highest value possible.

The source code of GO-MEP is made available at http://kiharalab.org/gomep for the academic community.

## Additional Information

**How to cite this article**: Chitale, M. *et al*. Missing gene identification using functional coherence scores. *Sci. Rep.* **6**, 31725; doi: 10.1038/srep31725 (2016).
